# Supplementary material for: Are carbon emissions trading and green financial instruments synergistic? -Comprehensive quantitative research based on content analysis
Source: PLoS One. 2024 Mar 7;19(3):e0298601. doi: 10.1371/journal.pone.0298601 (PMC10919601; doi:10.1371/journal.pone.0298601)
Supplement: S1 Data — (ZIP) [file pone.0298601.s001.zip › Data/地级市Z_F工作报告.zip!/─┌├╔╣┼/╬┌║ú╩╨/└┤╫╘╣1⁄2╓┌║┼í╛┬φ┐╦╩2╛▌═°í┐_2015.docx]

各位代表：

现在，我代表市人民政府向大会报告工作，请予审议，并请市政协委员和列席会议的同志们提出意见。

一、2014年工作回顾

2014年，面对严峻复杂的经济形势，市人民政府在自治区党委、政府和市委的正确领导下，在市人大、政协的监督支持下，牢牢把握稳中求进工作总基调，加快推进经济转型和城市转型，经济社会实现了持续稳步发展。预计全年地区生产总值完成620亿元，增长9%；固定资产投资完成488亿元，增长17%，提前一年完成“十二五”目标任务；公共财政预算收入完成75.16亿元，增长9.7%；社会消费品零售总额完成127亿元，增长12%；城乡常住居民人均可支配收入30433元，增长9.6%，其中，城镇常住居民人均可支配收入31538元，增长9.5%，农村牧区常住居民人均可支配收入13125元，增长10.5%。

（一）工业经济平稳发展，产业转型步入新阶段

积极应对经济下行压力，努力破解企业发展难题，强化经济形势研判调度，工业经济实现平稳运行。预计规模以上工业增加值增长11%；工业固定资产投资完成308亿元，增长21%。传统产业延伸提质。原煤利用水平不断提升，就地转化率达到90%以上。焦炉煤气年处理能力达30亿立方米，煤焦油深加工能力达到90万吨。天津亚东萘系深加工项目开工建设，神华硝铵、黑猫三兴二萘酚项目基本建成，宝化万辰煤焦油深加工一期试产，华油焦炉煤气制天然气二期建成投产，焦炉煤气实现高效利用。铁路运输结构进一步优化，“白货”发运比重由56%提高到68%。新兴产业比重不断提高。精细化工产业快速发展，腾龙生物精细化工二期建成投产，成城交大、佳瑞米精细化工部分产品建成试产。全市电力装机容量达到389万千瓦，增长8.1%，完成电力多边交易42亿度，为企业减少电费支出1亿元。东源科技2×35万千瓦、君正二期1×20万千瓦2个电厂项目获得国家批准。装备制造业发展迅速，陕汽清洁能源汽车产销量突破3000辆，有力带动了瑞格工贸、金运商贸等近20个配套项目发展。雅泰高科技产业园填补了我市医学工程领域空白。企业发展实力不断壮大，黄河能源、源通煤化进入中国民营企业500强，君正能源等8家企业进入内蒙古民营企业100强。园区承载能力不断提升。乌海经济开发区成功列入首批国家低碳工业园区试点，全国电石法聚氯乙烯产业知名品牌创建示范区获准筹建，开发区总体规划获自治区批复。“环乌海湖”国家级高新技术产业开发区申报进展顺利。海勃湾工业园区成为全国首批循环化改造试点园区，华资、源通、天宇化工成为自治区循环经济试点（示范）企业，我市被列入自治区煤电灰铝一体化循环产业示范基地发展规划。科技创新能力进一步增强。全社会研发经费投入占地区生产总值比重达到1.4，居自治区首位。全市自治区级企业研发中心达到14家、研究院6家。建立我市首个自治区院士专家工作站。非公经济持续健康发展。预计非公经济增加值完成420亿元，增长13%，占地区生产总值比重为69%，非公经济对固定资产投资贡献率达71%，拉动固定资产投资增长18个百分点。招商引资成效明显。大力实施精准化招商，全年实施招商引资项目233个，引进到位资金291亿元，增长7.4%，争取上级各类资金32亿元。

（二）城市功能逐步完善，区域中心城市彰显新魅力

城市规划体系不断健全。城市总体规划评估完成评审，资源枯竭城市转型规划获得自治区批复，滨河西区控制单元规划、滨河二期控制性详细规划和城市设计概念性方案编制完成，绿地系统规划、机场净空规划、海勃湾旧城区市政公用设施专项规划完成编制，环乌海湖旅游规划完成中期成果。环湖开发建设步入新阶段。国家重点项目黄河海勃湾水利枢纽工程完工并顺利蓄水，四台机组实现发电。滨河二期基础设施建设全面启动，四横三纵7条主干道及配套管网全部建成，环湖路14.1公里建成使用，主要道路框架初步形成。开元码头投入使用，库区右岸综合整治一期工程建成完工。城市夜景景观进一步提升，全年投入资金4500万元，重点实施了甘德尔山生态文明景区、环乌海湖等亮化工程。市政公用设施逐步完善，供热和燃气普及率均达到84%，生活垃圾无害化处理率和生活污水集中处理率分别达到90.7%和94.5%。旧城区人居环境得到改善，完成既有建筑节能改造64万平方米；积极推广绿色建筑，实施绿色建筑项目72.7万平方米。我市成为全国第七个住宅产业化综合试点城市。城市精细化管理进一步加强。全面启动全国文明城市创建等“六城联创”工作。制定了《黄河海勃湾水利枢纽管理办法》和《乌海湖水环境污染防治规划》，环湖管理得到加强。便民服务水平进一步提升，“12345”市民服务热线和数字城管监督指挥平台运行成效明显。我市被列为信息惠民国家试点城市。立体交通网络日趋完善。桌子山街公铁立交桥投入使用，海勃湾碱柜公交首末站主体完工，乌海湖大桥、110国道黄河大桥等重点项目顺利推进，荣乌高速乌海段、海勃湾生态涵养区道路工程、市客运枢纽站开工建设。新开通乌海至成都航线，乌海机场货邮吞吐量突破2000吨，旅客吞吐量46万人次，保持在全国民用机场旅客吞吐量百强行列。生态环境持续改善。完成坝址公园、书法广场、黄河风情园改造、葡萄主题公园、甘德尔山东坡等绿化工程，建成区新增绿地面积1075亩，绿化覆盖率达到41.9%，全年完成造林面积7万亩。城市水系建设稳步推进，甘德尔河一期、二期实现联合调度运行，高山草甸灌溉工程、桌子山矿区复绿引水工程（南线）全部完工。海勃湾北部生态涵养区建设控制规划编制完成，饮用水源地保护不断加强。环境治理力度加大。加快淘汰落后产能，全年淘汰电石29.4万吨、钢铁26.4万吨。节能减排扎实推进，实施减排项目76个，电力企业全部达标排放，所有水泥企业完成脱硝工程，节能减排完成自治区约束性指标，提前一年完成氮氧化物“十二五”减排任务。焦化行业提标改造加快实施，污染防治水平进一步提高。骆驼山矿区环境综合整治取得阶段性成果，完成煤矸石着火点压覆治理90%以上。集中整治环境污染突出问题，对13家环境违法企业实施停产治理，关停违法排污企业98家，淘汰燃煤小锅炉111台。加快环境管理信息化建设，主要企业污染物排放监控实现全覆盖。全年中心城区环境空气质量达标率63.7%。

（三）服务业比重进一步提高，第三产业发展迈出新步伐

预计全年第三产业增加值完成200亿元，增长7%。旅游业发展态势良好。金沙湾被评为国家沙漠公园，乌海湖列入国家旅游重点项目目录，乌海湖水上营运项目启动实施，甘德尔山生态文明景区、龙游湾湿地公园、农业休闲采摘园等旅游项目稳步推进，全年旅游总收入24.8亿元，增长22%，接待游客163.8万人次，增长12.5%。商贸流通业发展步伐加快。红星美凯龙开业运营，维多利广场一期基本建成，月星家居、万达广场主体封顶。鸿达物资交易中心投入使用，乌海北货场等物流项目积极推进。对外开放水平不断提高。乌海海关获得国家批准设立，承担一市三镇海关业务。对外贸易总额达到1860万美元，实现翻番。实际利用外资5831万美元。房地产业有序发展。全市房地产投资完成49亿元，增长1.8%，销售面积104万平方米。金融服务业持续稳定。全市银行存款余额600.4亿元，市内外贷款余额690亿元，分别增长2.2%和13.1%。金融机构贷款不良率2.57%，低于自治区平均水平。金融业对税收的贡献度达到8%。成功举办内蒙古西部盟市非公有制经济金融服务峰会，落实资金42.6亿元。

（四）“十个全覆盖”工程基本完成，新农区建设水平实现新提高

全年投入9.6亿元用于“十个全覆盖”工程，村村通电、安全饮水、校舍安全改造等8个方面基本实现全覆盖，农区危房改造完成2091户，街巷硬化完成50.6公里。农业产业化稳步推进，预计第一产业增加值完成5.6亿元，增长3%。全市销售收入500万元以上加工企业实现销售收入4.5亿元，规模以上流通企业8家，实现交易额20亿元。认真落实支农惠农政策，落实各项资金7249万元，帮助农业企业解决贷款5.2亿元。蔬菜、花卉苗木等优势产业发展壮大，蔬菜种植1.56万亩。葡萄产业发展迅速，出台了进一步加快葡萄产业发展的指导意见，新植补植葡萄8542亩，保有面积3万亩。汉森葡萄、葡萄酒通过欧盟有机认证，汉森公司上榜中国最具品牌价值500强，“云飞”葡萄获得第十二届中国国际农产品交易会金奖。

（五）民生得到持续改善，群众生活质量实现新提升

全年用于民生和社会事业支出70.68亿元，增长13.5%，占全市公共财政预算支出的74.6%。就业创业工作稳步推进。全市城镇新增就业8168人，完成就业创业培训9162人，2413人成功创业。城镇登记失业率3.25%，低于自治区控制目标。小额担保贷款额度进一步提高，全年发放小额担保贷款1.34亿元。社会保障体系逐步完善。社会保险覆盖面不断扩大，我市成为全国首批“全民参保登记计划”国家级试点城市。大病保险补偿比例、医疗保险住院和门诊报销比例均位居自治区前列，医疗救助“一站式”即时结算服务实现全覆盖。全市最低工资标准提高到每月1500元，企业退休人员养老金提高到月人均1819元。社会救助水平不断提高，最低生活保障标准由470元提高到570元，居自治区第1位。五保对象和“三无人员”实现同城同待遇，集中和分散供养标准由每人每年8000元、5000元，提高到18000元、13920元，分别居自治区第1位和第3位。孤儿集中抚养和分散抚养标准由每人每月1610元、1350元，分别提高到1700元、1418元，居自治区第3位。计划生育家庭奖扶标准提高到每人每年2000元，独生子女伤残死亡家庭扶助标准分别提高到每人每月1200元、1500元，居自治区第1位。免费孕前优生健康检查率达到92.3%，高于自治区目标值。保障性安居工程进展顺利。新开工建设各类保障性住房8973套，开工率102%，基本建成9633套。海勃湾依林佳苑社区获得自治区人居环境范例奖。重视老年人事业发展。出台了《老龄事业发展五年规划（2014-2018）》，老年养护中心及4个民办养老机构开工建设，建成老年人日间照料中心30个，13个社区居家养老试点启动实施。全力抓好“菜篮子”工程，群众“吃菜贵”问题逐步缓解。公交便民惠民政策力度加大，投入补贴资金1512万元，实施公交IC卡票价减免政策，乌海至碱柜、棋盘井、乌素图区域客运线路实现公交化运营。

（六）公共服务水平显著提升，社会事业发展开创新局面

教育事业协调发展。学前教育三年行动计划一期基本完成，共新建、改扩建幼儿园23所，幼儿入学规模新增6700个，“入园难”问题逐步缓解。投入学校标准化建设资金8000多万元，义务教育阶段办学条件不断改善。普通高中教学水平进一步提升，二本以上上线率58.4%，高于自治区平均水平14个百分点。市职业技术学校通过国家中等职业教育改革发展示范学校评估验收，乌海职业技能公共实训中心开工建设。医疗卫生服务水平进一步提升。积极探索分级诊疗模式，启动中医蒙医和妇幼保健两个医联体试点工作，基层医疗卫生机构服务水平不断提升。率先启动居民健康状况调查及全民健康促进五年行动规划，建成市级卫生数据中心，开通市民健康查询系统和区域妇幼保健信息系统，居民电子健康档案实现全覆盖。大力推广微创介入治疗技术，实施微创手术1412例。与北京大学肿瘤医院等国内知名医院开展合作，引入肿瘤放射治疗等新技术项目90余项，其中7项达到国家先进水平。我市被确定为自治区公立医院改革试点。文化体育事业繁荣发展。国内单体面积最大的当代中国书法艺术馆建成开馆，召烧沟岩画遗址保护一期基本完工。全国公共文化服务体系示范项目“书法五进”创建活动有序推进，广场文化艺术节等文化惠民活动广泛开展。满巴拉僧庙等5处文物被列入第五批自治区级重点文物保护单位。成功举办第六届黄河明珠·中国乌海书法艺术节暨第三届国际书法产业博览会、首届环乌海湖全国竞走挑战赛、第二届全国桥牌邀请赛等文体活动。足球改革发展工作全面启动，市足球协会和足球学校挂牌成立。社会治理工作稳步推进。深入开展平安社区、平安学校等“十小平安”创建活动，“平安乌海”建设成效明显。我市2个街道办事处、3个社区分别被评为全国和谐社区建设示范街道和示范社区。出台了支持社会组织发展的政策措施，社会组织焕发新活力。健全市、区、街道、社区四级群众工作网络，群众诉求表达渠道更加畅通，全市信访总量同比下降27.2%。推进“科技防控城市”建设，完善以校园、居民社区、公共场所、商业网点、重点单位和要害部位为主要内容的“六张技防网”。落实政府监管责任和企业主体责任，强化危险化学品、非煤矿山及冶金等重点领域安全专项检查，安全生产形势总体稳定。人口自然增长率为6.54‰，完成控制目标。支持工会、共青团、妇联等人民团体开展工作，关心残疾人工作，监察、审计工作得到加强，国防建设、双拥优抚、民族宗教、档案史志、外事侨务、人防、气象、防震减灾、新闻出版、广播影视、无线电管理和红十字会工作取得新成绩。

（七）改革创新积极推进，社会发展释放新活力

加快政府职能转变。加大政府购买社会服务力度，采购规模达4.32亿元，占同期采购总额的18.8%。推进扩权强县试点工作，行政效能进一步提高。土地确权登记颁证试点工作启动实施，农区居民土地承包经营权得到保障。行政审批制度改革不断深化。精简行政审批事项和行政事业性收费项目，缩减率分别达到55%和68%。建立全市统一的政务服务平台，审批流程不断完善，行政审批实现“一站式”服务。政府机构改革步伐加快。出台了《政府职能转变和机构改革方案》、《关于调整市、区工商、质监行政管理体制的实施意见》，完成工商、质监的调整和划转地方管理工作。推进食品药品监管体制改革，实现食品生产、流通、消费环节统一监管。深化市场准入制度改革。推进工商注册便利化，将注册资本实缴登记制改为认缴登记制，取消最低注册资本限制，有效激发了市场主体活力。工商登记制度改革实施以来，新增企业1214户，增长50.4%。

（八）依法行政水平不断提升，作风建设取得新成效

民主法制建设稳步推进。主动接受人大法律监督、政协民主监督，按时办结人大代表建议156件、政协提案101件。坚持邀请人大代表、政协委员列席市政府常务会议，广泛征求和吸纳各民主党派、各界群众和社会组织对政府工作的意见建议。深入推进“六五”普法，广泛开展“法律六进”活动，市民学法用法水平不断提高。政务公开深入推进，启用市、区、镇（街道办）三级政务公开网络平台，累计公开各类信息1.8万条。作风建设成效显著。党的群众路线教育实践活动取得阶段性成果，政府系统广大党员干部党性修养、宗旨意识、纪律观念得到加强，“四风”问题得到有效遏制。办公用房清理工作全面完成，公车改革稳步推进，处置超编车辆198辆。严控“三公”经费支出，全年压缩“三公”经费38%。“文山会海”得到有效整治，以市政府名义组织召开的会议减少14.2%，发文减少25.7%。政务协同办公平台建成试运行，初步实现办公自动化。

各位代表，过去的一年，我市经历了经济下行、外部环境复杂多变的严峻考验，全市经济社会发展稳中有进。这是自治区党委、政府和市委正确领导的结果，是市人大和市政协支持监督的结果，是全市各族人民奋力拼搏的结果。在此，我代表市人民政府，向全市广大干部群众和关心支持乌海发展建设的各界人士，表示崇高的敬意和衷心的感谢！

同时，我们也要清醒地认识到发展中存在的问题，主要是：产业结构不合理，传统产业抵御市场风险能力不足，新兴产业还处于培育发展阶段，初级和中低端产品比重依然较大，产业转型升级进程还需加快；资源环境与加快发展的矛盾不断加剧，水资源、矿产资源、土地资源瓶颈约束日益趋紧，大气污染防治任务仍然较重，矿区地质环境综合整治力度还需进一步加大，区域环境治理有待加强；公共服务优质资源不足，城市基础设施还不完善，民生工作还存在一些薄弱环节；政府与市场、社会的关系还没有理顺，公共服务、市场监管等职能发挥还不到位，一些群众关心的热点难点问题还没有得到根本解决。对于这些问题，我们将采取有力措施，认真加以解决。

二、2015年工作安排

2015年，政府工作的总体思路是：深入贯彻落实党的十八大和十八届三中、四中全会及中央经济工作会议精神，深入学习贯彻习近平总书记系列重要讲话和考察内蒙古重要讲话精神，全面贯彻落实自治区党委九届十一次、十二次、十三次全委会议各项工作部署和“8337”发展思路以及市委六届八次、九次、十次全委会议精神，牢牢把握稳中求进工作总基调，以提高经济发展质量和效益为中心，牢固树立持续发展、转型发展、协调发展、和谐发展理念，主动适应经济发展新常态，把稳增长、调结构、促改革、惠民生、保稳定贯穿发展全过程，加速经济转型和城市转型，加快打造自治区西部区域中心城市，努力构建富裕美丽文明和谐幸福乌海，打造祖国北疆亮丽风景线。

2015年，我们的主要预期目标为：地区生产总值增长8.5%，规上工业增加值增长11%，公共财政预算收入增长7%，全社会固定资产投资增长13%，社会消费品零售总额增长11%，城镇常住居民人均可支配收入增长9%，农村牧区常住居民人均可支配收入增长10%，节能减排完成自治区约束性控制指标，居民消费价格指数控制在3%左右，城镇登记失业率低于自治区平均水平。

这些预期目标的制定，充分考虑了经济发展新常态下，增速换挡与产业转型升级的双重要求，权衡了各种因素，并与编制“十三五”规划和全面建成小康社会目标相衔接，符合经济中长期发展规律，反映了现阶段我市经济增长潜力，是实事求是、积极稳妥的，也是需要经过艰苦努力才能实现的。围绕上述目标，要重点抓好以下六个方面工作：

（一）更加注重促进经济稳定增长，在发挥投资的拉动效应上求突破

继续发挥投资对稳增长的关键作用，扩大有效投资，保持投资强度，稳定企业生产，强化招商引资，实现经济持续平稳发展。

抓好重点项目建设。坚持增量调整方针，充分发挥大项目在转型升级中的龙头带动作用。继续实施重点项目领导包联责任制，突出抓好建成项目达产、续建项目推进、新建项目开工、前期项目审批、在谈项目落地。结合“十三五”规划编制，精心谋划一批事关发展全局的重大项目，积极争取列入国家和自治区计划盘子，为长远发展夯实基础。

稳企业促增长。强化宏观经济形势分析，围绕企业生产运行细化服务举措，及时制定出台有效的政策措施，帮助企业稳定生产、开拓市场，促进产能充分发挥。积极引导企业家转变发展观念，更加注重市场和消费心理分析，培育一批优秀企业家，充分发挥企业家才能。鼓励、引导大企业与中小企业通过产业配套联合协作发展，实现优势互补、市场共赢。进一步抓好自备电厂建设，积极参与蒙西电网电力体制改革，扩大电力多边交易范围，降低企业生产成本，增强市场竞争力。加大铁路运输协调工作力度，加强煤电油运等生产要素的平衡保障工作，稳步推进水权置换，缓解工业用水问题。进一步清理涉企收费，减轻企业税费负担。

转变投融资方式。大力推行政府与社会资本合作（PPP）模式，吸引社会资本参与基础设施、产业发展、社会事业、城乡建设、环境保护等重点领域建设。创新融资模式，以存量资产作股权、以建设任务引资金，与大企业开展深度合作。充分发挥政府投资的引导作用，整合资金，集中分类使用，由直接支持具体项目转化为设立投资基金，实行基金式项目化管理，最大限度发挥资金的使用效益。进一步激活土地、矿产资源的金融潜能，深入推进土地、矿业权抵押融资。准确把握国家宏观调控政策取向和投资方向，加强与上级部门对接，积极争取政策、项目、资金支持。

强化招商引资。充分发挥工业园区的招商引资载体作用，进一步完善综合配套服务功能，加大水、电、汽、热等基础设施建设改造力度，抓好“三废”处理设施建设，为入园企业搭建电子商务、金融等信息服务平台，切实增强园区承载力。修订招商选资标准，严格设定技术装备、安全节能环保、土地集约利用、投入产出等方面的准入条件。紧紧围绕煤焦化工和氯碱化工两大基地建设，瞄准国内外一流企业，依托现有产业基础，积极开展以商招商、产业链招商、产业集群招商、部门联动招商，探索实施代理招商、中介招商，加快引进一批转型升级优质项目。深入推进全领域、多层次招商，同步加大城市建设、社会事业、服务业等方面招商力度。继续优化政务环境，提高行政效率，强化跟踪服务，确保签约项目尽快落地，力争全年引进到位资金 300亿元以上。

（二）更加注重发展方式转变，在结构调整和优化上求突破

主动适应经济发展新常态，加快转变发展方式，着力调整优化产业结构、区域结构，全面提升经济发展质量和效益。

推动传统产业新型化。充分发挥煤焦化工、氯碱化工两大产业规模优势和成本优势，着力推进资源精深加工和产业链延伸，统筹推动煤、电、化及相关产业一体化发展，加快培育配套产业和下游产品，推动传统产业向中高端迈进。延伸发展煤焦化工，积极探索焦炭气化路径，鼓励焦炭企业围绕煤气制LNG、制甲醇等主导产业链，提高焦炭就地转化能力，逐步实现由以焦为主向以化产为主转变，摆脱对钢铁产业的严重依赖。拓展优化氯碱化工，重点推进PVC精深加工，大力发展延链补链项目，推动氯碱产业向集群化、精细化、高端化发展。

推动新兴产业规模化。强化龙头企业牵引作用，围绕陕汽清洁能源汽车带动镁轮毂、煤机、化机等装备制造产业向集群化发展，鼓励建设符合节能环保要求的高端铸造、锻造项目，重点抓好紫荆贝氏体钢、中钰镁合金轮毂、陕汽零部件配套产业园等项目建设。加快推进以多元醇、农药医药中间体等产品为重点的精细化工产业向规模化发展，鼓励生产染料、颜料等贴近终端消费市场的产品，重点抓好天津渤海精细化工、亚东萘系深加工、联合化学颜料等项目建设。全力推动以恒业成为中心的有机硅深加工、光伏制造等硅化工产业向高端化发展，形成产业竞争新优势。加快以政务云中心为基础的信息化基础设施和服务能力建设，探索发展云计算产业，夯实“两化”融合基础。积极发展新能源、新材料等非资源产业，着力培育新的经济增长点。

推动支柱产业多元化。培育壮大生产性服务业。着力发展现代物流、检验检测认证、工业信息服务、员工培训等现代服务业。积极培育和引进大型物流企业，重点发展铁路物流，加快建设乌海北货场及配套综合物流园、物流信息与大宗商品交易中心等项目，引导和支持发展第三方物流。培育发展职业教育产业，把乌海职业技能公共实训中心作为开展区域职业培训的平台，打造具有区域特色的精品专业和培训品牌。打造区域金融服务中心。加强金融服务体系建设，组建国有资产投融资公司，加强国有资产的资本化运作。积极争取全国性股份制商业银行、保险公司在我市设立区域性分支机构。加强金融风险防范，建立健全化解各类风险的体制机制，切实维护金融安全稳定。提升旅游业发展品位。高标准高起点推进环山湖建设，提升金沙湾、甘德尔山生态文明景区层次，加快龙游湾湿地公园建设。推动书法艺术、工艺美术等文化资源与旅游资源整合，开发具有地域特色和民族风情的旅游产品。加快完善旅游业配套服务功能，提高景区、酒店、旅行社旅游接待能力。加强与周边旅游线路的衔接，加大旅游品牌推介和宣传，着力打造中国西部旅游集散中心。促进商贸服务业快速发展。推动月星家居开业运营，加快维多利、九龙国际等项目建设，吸引高端业态、知名品牌进驻。制定出台电子商务发展扶持政策，加快电子商务应用平台建设，促进电子商务与传统商贸产业深度融合，提升商贸业水平。推进农业产业化。扶持和引进农业龙头企业，健全与农区居民的利益联结机制。转变农业生产经营方式，鼓励农区居民种植经济效益好、附加值高的蔬菜瓜果、花卉苗木。加大对葡萄产业的支持力度，继续引进葡萄新品种，年内新增葡萄3000亩以上，新建葡萄酒庄3座，提升“乌海葡萄”地理标志知名度，着力打造中国沙漠原生态葡萄酒庄品牌。切实抓好“菜篮子”工程，重点推动设施农业发展，年内种植蔬菜1.6万亩以上。加快一、二、三产相融互促，积极发展休闲采摘、旅游观光农业，培育自治区级休闲农业观光点2家。严格落实耕地保护制度，坚守耕地红线。积极稳妥做好土地承包经营权确权登记颁证试点工作，推进农区土地依法合理流转。

推动区域协调发展。积极配合落实《乌海及周边地区城镇规划》，立足服务区域，优化整合资源，聚集发展要素，强化功能配套，加快建设自治区产城融合示范区。大力发展市域经济，统筹“一区四园”发展布局，实现错位互补发展，提升地区经济核心竞争力。统筹城乡协调发展，按照城区现代化、农区城镇化、城乡一体化的发展思路，将城区和农区作为一个整体统筹规划，建立合理的城乡体系和空间结构。集中人力、物力、财力，高标准推进农区“十个全覆盖”，加快工程进度，提高工程质量，努力将团结新村、泽园新村、赛汗乌素村等示范村打造成自治区一流的样板工程。研究建立农区公共服务管理体制，探索综合执法向农区适度延伸，推进基础设施和公共服务城乡连接、共建共享。

（三）更加注重提升城市宜居水平，在功能完善和管理服务上求突破

坚持建管并重，高标准推进城市规划建设，把城市管理摆在突出位置，全力抓好生态建设和环境保护，努力打造服务功能完善、综合要素集聚、比较优势突出的自治区西部区域中心城市。

坚持高标准规划。启动城市总体规划调整报批，编制完成《乌海环山湖景区旅游总体规划》、《龙游湾湿地旅游总体规划》、《黄河乌海湖航运规划》和《城市公共交通规划》，完成城市转型发展战略研究和市政基础设施、消防、历史风貌保护等专项规划。重视各类规划的衔接与配合，积极探索“多规合一”。着力维护规划的权威性和严肃性，保证规划刚性落实。

积极推进城市建设。高标准推进滨河二期规划建设，完成环湖路桥、中央公园等项目建设，建成游船码头、游客服务中心、停车场等旅游配套设施，着力将“环乌海湖”区域打造成功能完善、环境优美、宜居宜业的城市核心区。有序推进旧城更新和改造，积极实施老旧管网改造，开工建设海勃湾东环路及地下管网工程，完成车站北路、建设北路续建工程，实施海南城区和工业园区热电联供。全力推动住宅产业现代化工作，完成既有建筑节能改造21.2万平方米。不断完善交通网络，完成乌海至陶乐公路改建、千里山与蒙西园区边界道路、海勃湾和乌达农业园区道路、110国道改扩建下穿包兰铁路工程，加快建设乌海湖大桥、110国道黄河大桥、荣乌高速乌海段、市客运枢纽站，开工建设乌达巴音赛互通立交桥、乌达公交枢纽站。

提升城市管理服务水平。以创建全国文明城市为统领，全面推动国家循环经济示范城市、全国水生态文明城市、国家卫生城市等“六城联创”工作。把科学化、人性化、制度化、精细化理念贯穿始终，从环境卫生、违章治理、便民摊点、广告设置等细节入手，建立标准统一、反应迅速、部门联动的城市管理机制。全力推动“智慧城市”和信息惠民国家试点城市建设工作，完善智慧乌海民生服务平台，建成区划地名地理信息系统，提高城市管理信息化服务水平。加强夜景景观亮化工作，加快环山环湖亮化建设，推广使用亮化设施智能控制系统。制定出台餐厨垃圾回收管理办法，完成乌达区、海南区生活垃圾无害化处理工程。培育引进专业化管理服务公司，切实提高城市管理服务效率和效能。优化公交路线和车辆配置，完善公共交通服务水平。

扎实推动环境保护和节能减排。认真贯彻新《环保法》，组织实施《乌海及周边地区大气污染防治规划》，积极推进区域大气污染联防联控，重点加大城区烟尘、粉尘、扬尘综合整治力度，建成区10蒸吨及以下燃煤锅炉全部淘汰。加强城区、农区、工矿区和交通沿线环境整治，加快矿区地质环境综合治理，全面启动市、区两级矿区地质环境综合整治示范工程。积极开展排污权有偿使用和交易试点工作，探索建立环境污染市场化管理。加大环境执法监察力度，坚决打击偷排漏排等环境违法行为。建成乌达区、海南区PM2.5空气质量自动监测站。大力淘汰落后产能，全年淘汰炼铁110万吨、水泥60万吨、电石7.5万吨、铁合金4.2万吨。鼓励发展循环经济和清洁生产，推进重点用能企业节能技术改造工程。加快实施焦化行业提标改造工程，实现全市焦化企业达标排放。年内实施减排项目97个，全面完成“十二五”主要污染物减排任务。启动市固废处理厂建设，大力推动煤矸石、粉煤灰、化工废渣等工业固废综合利用。提高园区污水处理和再生水利用水平，建成海勃湾和海南工业园区污水处理厂，确保乌达工业园区污水处理厂实现中水回用。认真落实《关于加强海勃湾北部生态涵养区保护和管理的实施办法》，加强地下水源地保护和乌海湖水质的监测工作，确保水环境安全。

大力推进生态建设。认真落实最严格水资源管理制度，做好水生态文明试点城市中期验收工作，加快推进甘德尔河三期工程建设。加快国家森林城市创建工作，年内建成国家园林城市。积极实施乌海湖右岸、甘德尔河南岸、环湖路、包兰铁路沿线等绿化工程，重点做好龙游湾湿地公园绿化、摩尔沟矿区植被恢复治理、海惠公路两侧绿化、乌达至海南城际快速通道绿化提升等工程，积极抓好村庄、老旧小区绿化。强化成本意识，坚持适地适树原则，合理选择树种，加大苗木自给，建成低碳产业园区苗圃基地和滨河西区育苗基地。加大义务植树动员宣传力度，培育壮大本地绿化队伍，进一步降低绿化建设管理运营成本。转变绿化投资方式，吸引更多社会资金参与绿化。大力推进京津风沙源治理、天然林保护等国家重点林业工程，年内完成绿化面积7万亩。

（四）更加注重创新驱动，在深化改革开放上求突破

坚持以改革创新为动力，以扩大开放促转型，不断破除各方面体制机制障碍，充分激发经济增长活力。

积极推进重点领域改革。厘清政府与市场关系，建立行政权力清单、市场准入负面清单和监管清单制度，全面公开政府部门工作流程、时限和责任。认真落实《政府职能转变和机构改革方案》，完成政府机构整合。理顺城市建设管理体制，推动市直部门部分管理职能下放。完善公共服务供给模式，扩大推行政府购买公共服务范围。深化行政审批制度改革，清理行政审批事项和行政事业性收费项目，优化并联审批程序，进一步提高行政效率。规范经营性服务收费，加大对各种乱收费行为的监管力度。深化国库集中收付制度改革，实现财政资金集中支付全覆盖，完善一般性转移支付增长机制。加快社会信用体系建设，加大失信行为惩处力度。

增强自主创新能力。大力推动企业技术进步和全面创新，利用好企业研发中心和研究院平台，加快推进一批关键共性技术的科研攻关，着力推动科技成果转化。加强企业与科研院所的产学研合作，开展新材料、新能源、节能环保领域的应用技术研究。坚持质量立市，加快自治区氯碱化工产品质量检验中心建设。充分发挥院士专家工作站作用，加大专业人才培养力度，推动城市建设管理与网络通讯技术深度融合。

提高对外开放水平。抓住国家建设丝绸之路经济带、自治区深化对俄蒙开放合作的战略机遇，着力将我市建成蒙古国煤炭落地加工基地。完成乌海海关筹建，建成海关出入境检验检疫监管区，组织开展通关业务和对外贸易业务培训，为外贸经济发展提供有力保障。加强海峡两岸文化体育、教育、医疗卫生交流合作，进一步提高我市的对外知名度。

（五）更加注重民生持续改善，在提高公共服务水平上求突破

坚持“守住底线、突出重点、完善制度、引导舆论”的基本思路，下大力气解决事关群众切身利益问题，努力为全市人民提供更优质的创业就业环境、更可靠的社会保障、更舒适的居住条件、更高质量的教育、更高水平的医疗卫生服务、更丰富多彩的文化生活、更和谐稳定的社会环境。

加大民生工作力度。推进就业创业工程。把就业作为民生工作的重中之重，多渠道开发就业岗位，积极做好高校毕业生、库区移民、退役军人就业。加大小额担保贷款扶持创业力度，加强创业园和创业孵化基地建设，着重发挥创业带动就业倍增效应。大力发展劳动密集型产业、服务业和中小微企业，实施援企稳岗政策，进一步稳定和扩大就业。加大就业困难人员社保补贴力度，对困难家庭、失业人员实施就业援助帮扶，动态消除零就业家庭。全年新增城镇就业7500人，失业人员再就业3000人，完成培训7900人次以上，发放小额担保贷款1亿元以上。提升社会保障水平。推进“全民参保登记计划”试点工作，重点抓好高风险行业、中小微企业、灵活就业人员的社会保险扩面工作。健全完善医疗保险政策体系，利用信息化管理手段推进医保付费方式改革，力争医保基金收支平衡。按照国家、自治区统一部署，适时推进行政事业单位养老保险制度改革。加快推进“市民扶贫脱困工程”，实现精准脱贫。完善社会救助制度，建立部门联动机制。推动养老服务产业发展，鼓励社会力量兴办养老机构，全面推进社区居家养老服务，建成市老年养护服务中心。完成全市殡葬事业发展规划。加快推进百姓安居工程。抓好乌达煤矿棚户区改造二期工程，加快实施海勃湾北站、新桥南社区和东山城市棚户区改造，完成海南老石旦矿区搬迁安置，积极做好老旧小区、城乡结合部社区、独立工矿区改造，新建各类棚户区改造安置房12832套，基本建成各类保障性住房7048套。实施公租房和廉租房并轨运行管理，探索建立建设资金回收机制，积极推进棚改货币化安置，建立向存量市场购买公租房、廉租房、棚户区改造安置房的住房保障新模式。

推动社会事业全面发展。提高教育教学质量。实施第二期学前教育三年行动计划，加强优质公办幼儿园建设，加大对民办幼儿园扶持力度。推动义务教育均衡发展，提升义务阶段办学水平。深化高中课程改革，优化教育教学管理，提升高中教育办学质量。制定现代职业教育体系发展规划，建立以技术培训为核心、与产业发展需要相配套的课程体系，增设医疗护理、旅游、电子商务、物联网技术等专业，大力培养实用型人才。规范发展民办教育，加快发展民族教育、特殊教育。提升医疗服务水平。编制完成全市医疗卫生事业发展规划和健康服务产业发展规划。加快推进市人民医院改革试点工作。加强医疗卫生基础设施建设，加快市职业病防治院、市精神卫生中心、蒙中医院二期、残疾人康复中心项目建设，做好健康产业园区前期规划，开展市公共卫生综合服务中心前期工作。加强市妇幼保健院品牌建设，建成辅助生殖中心。整合全市医疗资源，以多学科推广微创技术为突破，提升医疗服务能力。加强医疗合作交流和人才队伍建设，大力引进专业技术骨干和学科带头人。建设覆盖城乡的区域卫生信息化系统，实现全市医疗信息资源共享。提高文体服务质量。深化文化管理体制改革，加快推动政府部门由办文化向管文化转变。创新完善当代中国书法艺术馆运营管理机制，建成乌海博物馆。加强公共文化服务体系建设，完成“书法五进”全国公共文化服务体系示范项目创建工作，扩大广场文化艺术节、文化活动进万家等惠民文化品牌活动影响力。积极发展文化产业，加大对文化产业的扶持力度，培育一批具有地方特色的中小微文化企业。全面推进足球改革工作，促进竞技体育和群众体育协调发展。

健全社会治理机制。认真落实《乌海市关于进一步加强和谐社区建设的意见》，完善社区服务中心规划设置。健全社区管理体制，理顺政府部门、街道办事处和社区关系，建立政府指导协调、居民自治管理、社会组织广泛参与的社区管理新常态。加大社区工作人员业务培训和绩效考核力度，建立与社区岗位相适应的薪酬机制。加强物业服务管理，积极探索社区、物业服务企业、业主三方共管共建新模式。严格贯彻实施新《安全法》，落实安全生产责任制，加快建设安全生产隐患动态监管与风险防御信息化系统，强化岗位安全操作技能培训，防范和遏制重特大安全事故发生。建立健全农产品质量和食品安全监管机制，加快建立食用农产品质量安全追溯体系，严厉打击食品药品违法犯罪。开工建设市水质检测中心、海勃湾区净水厂，启动乌达区和海南区净水厂建设，确保群众饮水安全。全力做好信访维稳工作，健全矛盾纠纷排查化解机制，畅通群众诉求表达渠道。严厉打击各类违法犯罪活动，保护人民生命财产安全，提高人民群众安全感。构建立体化治安防控体系，建设“平安乌海”。全面评估总结“十二五”规划实施情况，开展“十三五”规划编制，抓好自治区成立70周年和建市40周年迎庆筹备工作。继续做好计划生育工作，加强出生缺陷防治和出生人口性别比综合治理，促进人口均衡发展。加强民族宗教工作，促进社会和谐。充分发挥工会、共青团、妇联等人民团体的桥梁纽带作用，关心支持妇女儿童、红十字会、慈善救助等事业，进一步加强国防建设、双拥优抚、人民防空、气象、地震、档案史志、新闻出版、广播影视、无线电管理等工作。

（六）更加注重依法行政，在提高政府服务能力、加强廉政建设上求突破

建设法治政府。坚持依法履职，制定出台全面推进依法行政、加快建设法治政府的意见。切实发挥政府法制部门职能，健全行政决策合法性审查制度和风险评估制度，全面清理不适应发展需要的制度和文件。充分发挥律师顾问团作用，建立律师参与政府重大项目、重点工作决策论证制度，提高依法行政水平。加快推进行政执法体制改革，组建综合行政执法局。建立健全行政裁量权基准制度，加强行政执法信息化建设和信息共享，提高执法效率和规范化水平。完善法律援助和司法救助制度，维护群众合法权益。主动接受市委领导，自觉接受人大和政协监督，认真办理人大代表建议意见和政协委员提案，充分听取民主党派、工商联、无党派人士的意见。深入推进“六五”普法，增强全社会法治意识，引导群众自觉守法、遇事找法、解决问题靠法，着力建设法治乌海。

建设服务政府。推动领导方式和工作重点转变，加快经济建设型政府向公共服务管理型政府转变，更加注重民生和社会事业发展，更加注重资源节约型和环境友好型社会建设。认真贯彻中央“八项规定”，巩固党的群众路线教育实践活动成果，持续抓好“四风”突出问题整改，形成作风建设长效机制。健全领导干部带头深入基层调查研究制度，形成一批高质量的调研成果。厉行节约反对浪费，严控公务活动开支，推进公务用车制度改革，确保“三公”经费只减不增。提高公共服务部门办事效率，集中解决群众普遍关心的空气质量、物业管理等问题，提升群众满意度。理顺“12345”市民服务热线管理机制，进一步规范工作流程，提高为民服务质量。扩大政务公开内容和范围，健全公众参与行政决策机制。

建设廉洁政府。加强思想政治理论武装，严格执行党的纪律，始终在思想上政治上行动上与中央、自治区和市委保持高度一致。强化政府系统各级党组织建设，严格落实“一岗双责”，切实履行好党建主体责任，以党的建设推动科学发展。加强纪律建设，把严明政治纪律和政治规矩摆在更加重要的位置，深入推进党风廉政建设。加强廉政风险防控，严格执行“三重一大”事项集体决策制度，重大问题决策、重大项目投资和大额财政资金使用必须经集体讨论决定。深入推进执法纠风专项治理，严肃查处工程建设招投标、经营性土地招拍挂、政府集中采购等重点领域的腐败问题。加强行政监察和审计监督，坚决查处损害群众利益的不正之风。

各位代表，改革开启新征程，时代赋予新使命。让我们在市委的正确领导下，紧紧依靠全市人民，进一步凝聚改革发展稳定的正能量，锐意进取，真抓实干，为建设富裕、美丽、文明、和谐、幸福乌海而努力奋斗！
